# Supplementary material for: The ClpXP protease and the ClpX unfoldase control virulence, cell division, and autolysis in Streptococcus pneumoniae
Source: Microbiol Spectr. 2025 May 23;13(7):e00804-25. doi: 10.1128/spectrum.00804-25 (PMC12211083; doi:10.1128/spectrum.00804-25)
Supplement: Supplemental figures — Fig. S1 to S4. [file spectrum.00804-25-s0001.pdf]

Supplementary:

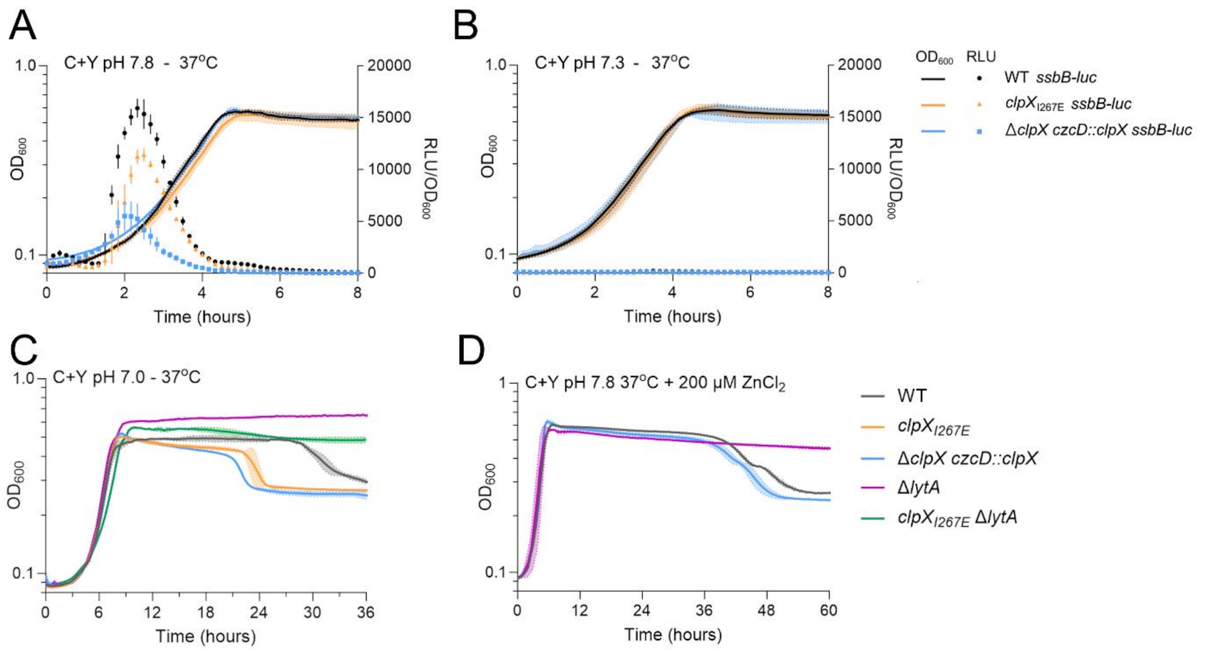

Figure S1| Competence is not induced at pH 7.3 for ClpX(P) mutated strains. *Growth of WT ssbB-luc (black), clpX$_{I267E}$ ssbB-luc (orange) and ΔclpX czcD::clpX ssbB-luc (blue) in C+Y with 0.5 mg/mL luciferine, at* ***(A)*** *pH permissive for competence (pH 7.8) and* ***(B)*** *non-permissive for competence (pH 7.3). Solid lines depict OD$_{600}$ values while solid symbols indicate RLU signal normalized to the OD$_{600}$ value.* ***(C)*** *Strains of WT (grey), clpX$_{I267E}$ (orange) ΔclpX czcD::clpX (blue), ΔlytA (purple), clpX$_{I267E}$ ΔlytA (green) grown in non-competence permissive C+Y pH 7.0 at 37°C for 36 hours.* ***(D)*** *Strains of WT (grey), ΔlytA (purple) and ΔclpX czcD::clpX (blue) were grown in C+Y media at 37° C with the addition of 200 µM ZnCl$_2$ for 60 hours.*

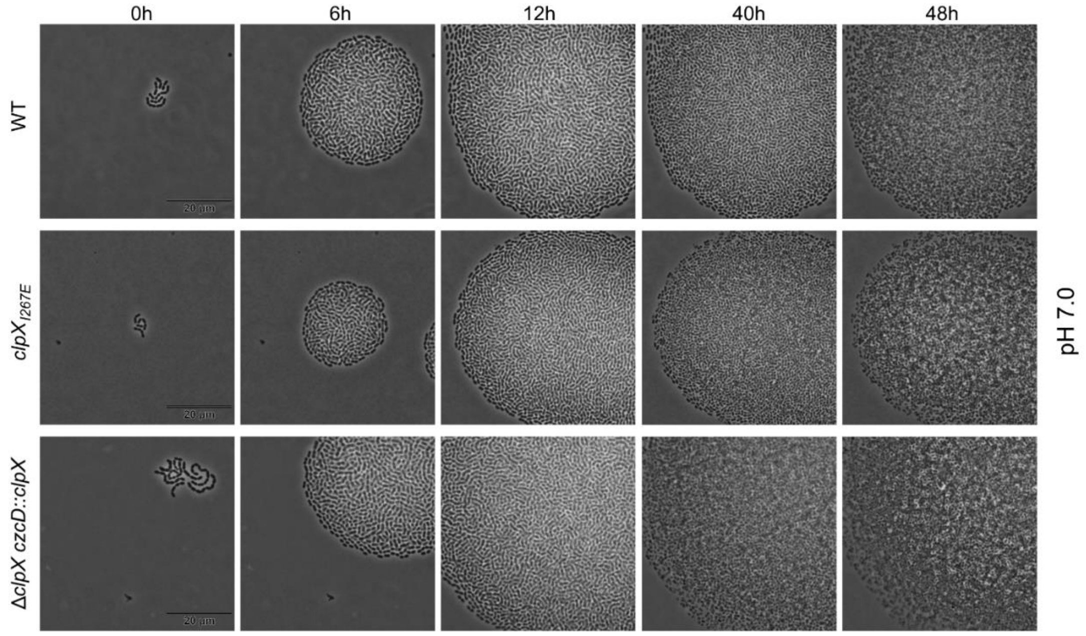

Figure S2| Strains grow mainly as diplococci in C+Y non-permissive for competence (pH 7.0). *WT, clpX$_{I267E}$ and ΔclpX czcD::clpX grown on pH 7.0 C+Y + 1.2% agarose at 37° C and phase-contrast imaged every 5 minutes.*

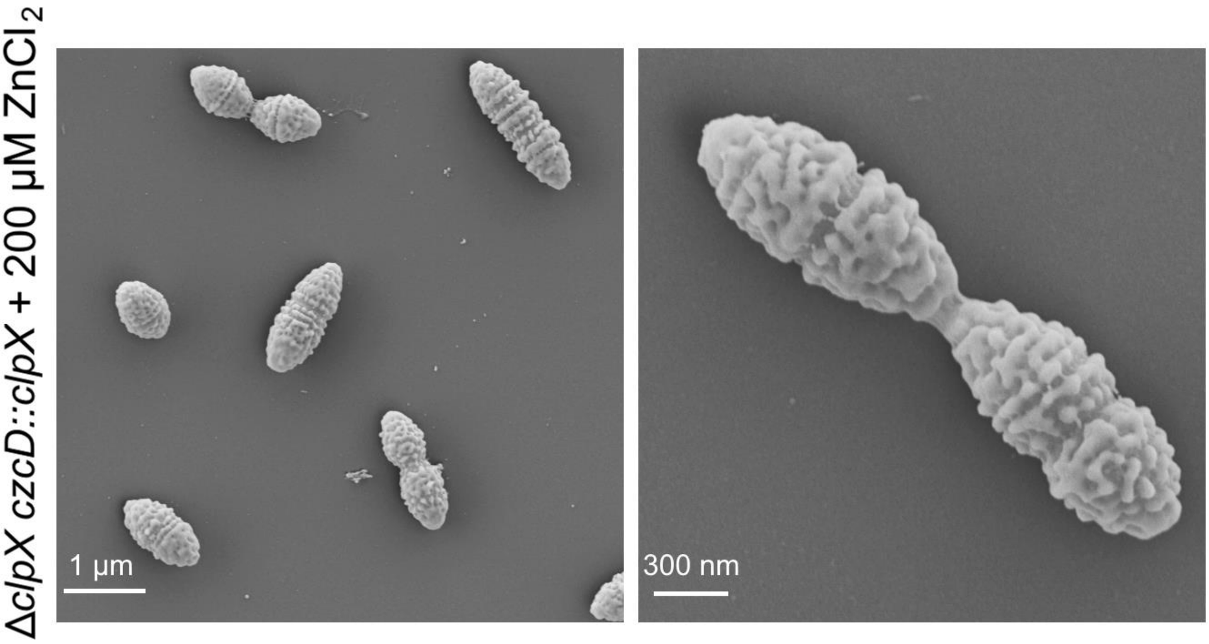

Figure S3| Addition of 200 µM $ZnCl_2$ restores the ΔclpX czcD::clpX strain to normal. *SEM images of the ΔclpX czcD::clpX strain grown in RPMI + 0.25 yeast extract + 200 µM $ZnCl_2$*

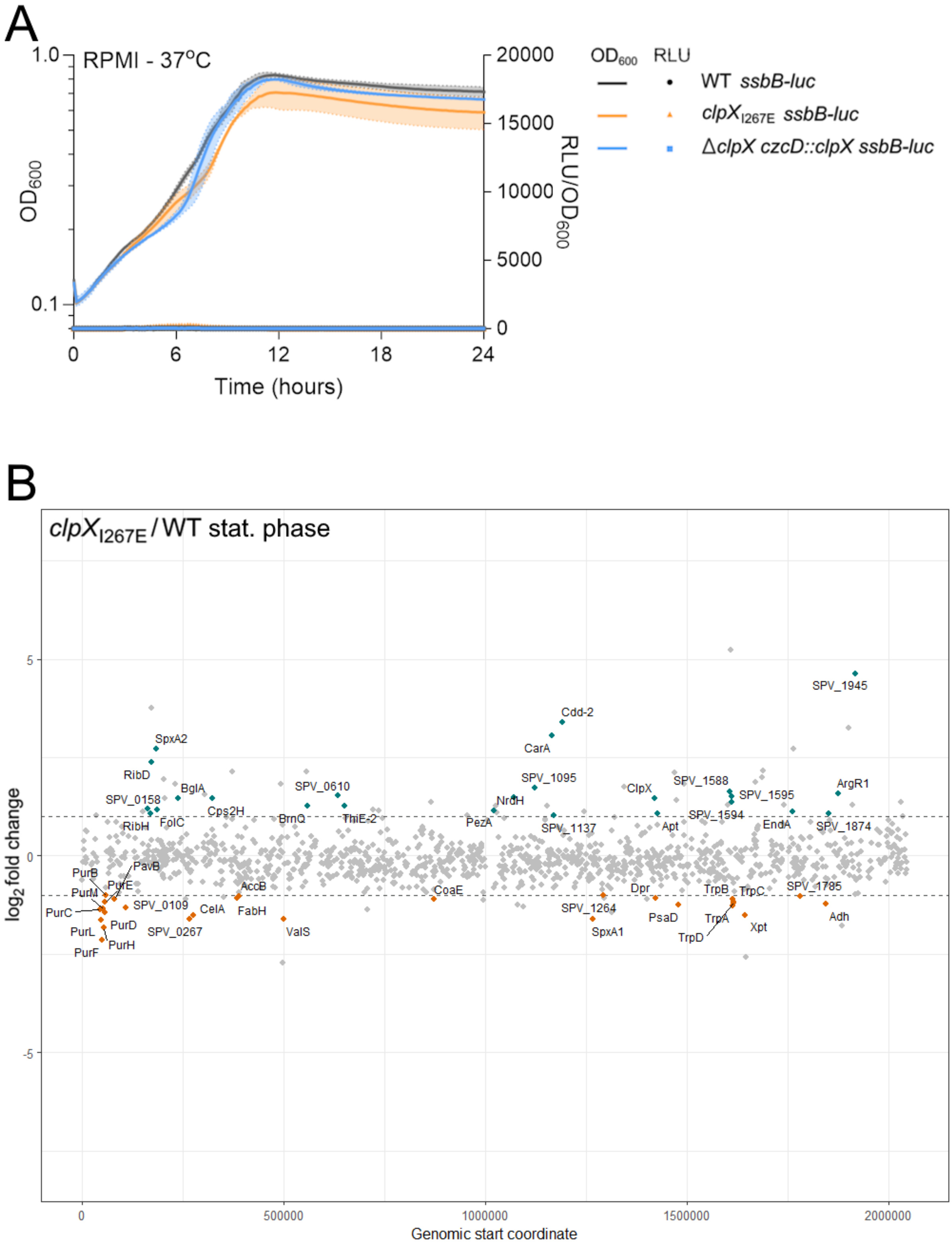

Figure S4| The clpX$_{I267E}$ proteome changes at stationary phase (OD$_{600}$ ~ 1.5) in the non-competence permissive media RPMI. (A) *Growth of WT ssbB-luc (black), clpX$_{I267E}$ ssbB-luc (orange) and ΔclpX czcD::clpX ssbB-luc (blue) in RPMI with 0.5 mg/mL luciferine at 37°C. Solid lines depict OD$_{600}$ values while solid symbols indicate RLU signal normalized to the OD$_{600}$ values.* ***(B)*** *Proteins (≥ 2 identified peptides) were quantified for clpX$_{I267E}$ relative to the proteome of the WT. Each protein is plotted with a solid dot, according to their genomic start coordinate (x-axis), in relation to their relative log$_2$ transformed fold change. Upregulated proteins (log$_2$ fold change > 1) with an FDR < 0.05 were given a green-blue color and labeled accordingly. Downregulated proteins (log$_2$ fold change < -1) with an FDR < 0.05 were given an orange color and labeled accordingly. Non-significant and/or non-changing proteins were plotted with a grey dot.*
