# Supplementary material for: The ClpXP protease and the ClpX unfoldase control virulence, cell division, and autolysis in Streptococcus pneumoniae
Source: Microbiol Spectr. 2025 May 23;13(7):e00804-25. doi: 10.1128/spectrum.00804-25 (PMC12211083; doi:10.1128/spectrum.00804-25)
Supplement: Table S2 — Settings used in microscopy. [file spectrum.00804-25-s0003.pdf]

Table S2

A. *Reversed phase liquid chromatography (RPLC)*

Instrument	Ultimate 3000 RSLC (Thermo Scientific)
Trap column	75 µm inner diameter, packed with 3 µm C18 particles (Acclaim PepMap100, Thermo Scientific)
Analytical column	Accucore 150-C18, (Thermo Fisher Scientific)<br>25 cm x 75 µm, 2,6 µm C18 particles, 150 Å pore size
Buffer system	binary buffer system consisting of 0.1% acetic acid in HPLC-grade water (buffer A) and 100% ACN in 0.1% acetic acid (buffer B)
Flow rate	300 nl/min
Gradient	linear gradient of buffer B from 2% up to 25%
Column oven temperature	40° C
Gradient duration	90 min:<br>0 min-2% B<br>2 min-5% B<br>10 min-7% B<br>70 min-25% B<br>75 min-40% B<br>77 min-90% B<br>83 min-90% B<br>85 min-2% B<br>90 min-2%B

B. *Mass spectrometry*

Instrument	Exploris 480 mass spectrometer
Electrospray	Nanospray Flex Ion Source
Operation mode	data-independent
Full MS	
MS scan resolution	120000
Norm. AGC target (%)	300
maximum ion injection time for the MS scan	60 ms

Scan range	350 to 1200 m/z
RF Lens	50 %
Spectra data type	profile
dd-MS2	
Precursor mass range	350 to 1200 m/z
Resolution	30,000
Norm. MS/MS AGC target (%)	3000
Maximum ion injection time mode	auto
Spectra data type	profile
Microscans	1
Isolation window	66 windows, 13 m/z, 2 m/z overlap (90 min)<br>50 windows, 17 m/z, 2 m/z overlap (75 min)
Define first mass	200
Dissociation mode	higher energy collisional dissociation (HCD)
HCD normalized collision energy	30%

C. Spectronaut^{TM} parameters used for analysis of mass spectrometry data .

Parameter	**Setting**
Identification	
Pvalue Estimator	Kernel Density Estimator
Precursor Qvalue Cutoff	0.001
Quantification	
Precursor Filtering	Identified (Qvalue)
Imputation Strategy	Use Background Signal
Quantity MS Level	MS2
Quantity Type	Area
Cross-Run Normalization	True
Normalization Strategy	Global Normalization (Median)
Row Selection	Identified in at least 1 Run (Sparse)
